# Epidemiological Investigation and Etiological Analysis of a Cutaneous Anthrax Epidemic Caused by Butchering Sick Cattle in Guizhou, China

**DOI:** 10.3389/fpubh.2020.00065

**Published:** 2020-03-25

**Authors:** Shijun Li, Qing Ma, Hong Chen, Ying Liu, Guanghai Yao, Guangpeng Tang, Dingming Wang

**Affiliations:** ^1^Laboratory of Bacterial Disease, Experiment Center, Guizhou Provincial Center for Disease Control and Prevention, Guiyang, China; ^2^Guiyang Centre for Animal Disease Control and Prevention, Guiyang, China; ^3^Guizhou Provincial Center for Disease Control and Prevention, Institute for Communicable Disease Control and Prevention, Guiyang, China

**Keywords:** anthrax, *Bacillus anthracis*, PCR, multiple-locus variable-number tandem repeats analysis (MLVA-15), single-nucleotide polymorphism (SNP)

## Abstract

A suspected human cutaneous anthrax epidemic caused by butchering sick cattle occurred in Zhijin County of Guizhou Province, Southwest of China, in 2016. Epidemiological investigation and etiological analysis were performed to provide a scientific basis for the source tracking of the epidemic. The epidemic was epidemiologically investigated, and skin blister samples collected from patients and soil samples collected from the butchering spots were used for *Bacillus anthracis* isolation. The suspicious *B. anthracis* isolates were identified using conventional methods and PCR, followed by genotyping using multiple-locus variable-number tandem repeats (VNTRs) analysis (MLVA-15) and canonical single-nucleotide polymorphism (canSNP). The genetic relationship of epidemic strains and isolates collected from other regions was analyzed. Epidemiological investigation results showed that the patients may be infected by *B. anthracis* during butchering sick cattle. Two suspected *B. anthracis* strains were isolated from blood samples and blister fluids, respectively. Conventional methods identified the two suspected isolates as *B. anthracis*, while PCR results showed that anti-protective antigen (PA) and capsule (CAP) gene were positive in the two isolates. MLVA-15 showed that the MLVA profiles of the two isolates were 9-20-12-53-16-2-8-8-8-4-4-4-4-10-4, which is different from the MLVA profiles of representative strains from other regions. CanSNP analysis showed that the two strains belonged to cluster A.Br.001/002. Clustering analysis and minimum spanning tree (MST) demonstrated that the two isolates were clustered with strains previously isolated from Guizhou Province. The results indicated that *B. anthracis* was the pathogen for this epidemic, and the patients were infected during butchering the sick. The genetic characteristics and the relationship of the *B. anthracis* isolates to strains from other regions indicated that the epidemic was a local occurrence.

## Introduction

Anthrax is a zoonosis caused by the pathogenic bacterium *Bacillus anthracis* (1). When nutrients are exhausted, *B. anthracis* can form a resistant spore that can survive for decades in soil. The natural hosts, herbivores, can be infected by contact with spore-contaminated soil. Humans can usually become infected when they touch animals infected by *B. anthracis* or products of infected animals (2). When endospores of *B. anthracis* enter the body by ingestion or inhalation, or through skin abrasions, the fatal anthrax often occurs (1, 3).

Humans commonly suffer anthrax by handling infected animals, carcasses of animals, or animal products (4). In addition, anthrax is a potential agent of bioterrorism and biowarfare, as spores of *B. anthracis* can be stable for decades and can be readily used as biological weapons (5).

*B. anthracis* is most closely related to *Bacillus thuringiensis* and *Bacillus cereus*, which belongs to the *B. cereus* group (2). The plasmids pXO1 and pXO2 are the major virulence factors of *B anthracis*. The plasmid pXO1 codes for lethal factor, edema factor, and protective antigen, which cause hemorrhage, edema, and necrosis, respectively. The plasmid pXO2 encodes cap B, cap C, and cap A gene, which is involved in the polyglutamyl capsule synthesis. Both plasmids pXO1 and pXO2 are necessary for the full virulence of *B. anthracis*, and loss of either leads to an attenuated strain (2). Anthrax infects humans through the skin, respiratory tract, or the gastrointestinal tract, which results in the cutaneous, inhalational, or gastrointestinal form of the disease, respectively (1). Cutaneous anthrax is the most common form of anthrax, which is caused by bacterial spore contamination of the skin with abrasion or damage (6).

It has been reported that an anthrax-like disease has occurred in China more than 5,000 years ago, and the symptoms and epidemiology of anthrax had been depicted (7). The results of an anthrax surveillance and control project that had been carried out in 10 provinces of China between 1990 and 1994 have been reported in 1995 (7). A more recent report showed that the distribution geographically declined overall from west to east, but the cumulative number of counties affected by anthrax ascended from 2005 to 2014. About 86% of anthrax cases took place in farmers or herdsmen, which indicated the shift of disease from industrial workers to farmers (8). Guizhou Province is one of the historical centers of anthrax in the southwest of China. A total of 17,975 cases of anthrax were reported in Guizhou Province from 1957 to 1999 (9), and a total of 32 outbreaks of anthrax occurred in Guizhou Province from 2001 to 2003 (10).

In September 2016, a suspected human cutaneous anthrax epidemic occurred in Zhijin County, which was reported to Guizhou Provincial Center for Disease Control and Prevention (CDC), and the suspected anthrax epidemic was then epidemiologically investigated. To provide an etiologic basis for the diagnosis of patients and control of the epidemic as well as to enhance our understanding of the genetic characteristics of *B. anthracis* in Guizhou Province, the skin blister and blood samples collected from patients and the soil samples collected from butchering locations were used for *B. anthracis* isolation. The isolates were identified using conventional methods and PCR and were subsequently genotyped using multiple-locus variable-number tandem repeats (VNTRs) analysis (MLVA-15) and canonical single-nucleotide polymorphism (canSNP). The genetic relationship between the epidemic strains and isolates collected from other regions was described in this study.

## Methods

### Epidemiological Investigation

In September 2016, a case of suspected anthrax infection was reported to Guizhou Provincial CDC. The field epidemiological investigation team investigated the epidemic and attempted to control the disease together with staff from the local county CDC. All suspected cases, exposed to tissues of the sick cattle, were investigated. These cases included anyone who was involved in the slaughtering, skinning, handling of meat and other tissues, or eating the meat of the sick cattle (11). All of the suspected human anthrax cases were diagnosed based on the diagnostic criteria for anthrax from the Chinese Ministry of Health (8).

### Bacterial Isolation

Human samples including blister fluid and blood were collected in the hospital. Soil samples were collected from the butchering locations. The above collected samples (Table 1) were used for *B. anthracis* isolation. Blister fluid was inoculated on blood agar plates or nutrient LB agar plates, followed by 24 h of incubation at 37°C. Soil samples were suspended in autoclaved saline and heated for 20 min at 60°C. The supernatants were inoculated cultured on the blood agar plates or nutrient LB agar plates and incubated for 24 h at 37°C. The suspected *B. anthracis* colonies were used for further ecological identification.

**Table 1:** Information of the samples used in this study.

**No**.	**Sample type**	**Number of samples**	**Source**	**Country**	**Prefecture**	**County**
1	Blister fluid	1	Patient	China	Bijie	Zhijin
2	Blood	1	Patient	China	Bijie	Zhijin
3	Soil	6	Environment	China	Bijie	Zhijin

### Conventional Identification of Bacterial Strains Suspected to Be *B. anthracis*

The suspected *B. anthracis* strains isolated from blood and blister fluid were inoculated onto new nutrient LB agar plates or blood agar plates. Conventional methods, including Gram staining, a phage lysis test, and a penicillin inhibition test for anthrax diagnosis, were applied to identify the suspected *B. anthracis* strains.

### DNA Template Preparation

Extraction of DNA from suspected *B. anthracis* isolates was performed using the boiling method (5). Briefly, *B. anthracis* isolates were inoculated onto blood agar plates and cultured for 20 h at 37°C. The bacterial colony was picked and added into a microcentrifuge tube containing 100 μl of DNA extraction liquid (Liferiver, China). The bacterial suspension was heated for 20 min at 95°C followed by cooling to room temperature. The suspension was centrifuged for 5 min at 15,000 g. The supernatant containing DNA was utilized as the template of PCR.

### PCR Identification

Identification of virulent isolates was performed by detection of the anti-protective antigen (PA) or capsule (CAP) gene of pathogenic *B. anthracis* (12). Amplifications were performed in 25 μl PCR reactions, which contained 12.5 μl of PreMix (TaKaRa, Otsu, Japan), 1.0 μl of PA or CAP primer, 9.5 μl of deionized water. Amplification was conducted with a Biometra TProfessional Thermocycler (Biometra, Goettingen, Germany) using the following parameters: 95°C for 5 min, followed by 30 cycles of 95°C for 1 min, 55°C for 1 min, and 72°C for 1 min. PCR products were detected by electrophoresis of 1 μl of amplified DNA using 1.2% agarose gel at 100 V for 30 min.

### Multiple-Locus Variable-Number Tandem Repeats Analysis

The MLVA-15 was conducted as previously reported (13, 14). The primers for the 15 VNTRs (Table 2) were described in a previous study (13, 14). Modifications to the capillary electrophoresis (CE) protocol are outlined below. HEX fluorescent dye was labeled on the forward primers (Table 2). Amplifications were performed in a 50-μl PCR system that included 25 μl of PreMix Taq (TaKaRa, Otsu, Japan), 2 μl of each forward and reverse primers (10 pmol/μl), 2 μl template of DNA, and 19 μl of deionized water. PCR amplification was conducted with Biometra TProfessional Thermocycler (Biometra, Goettingen, Germany) according to the following parameters: 94°C for 5 min (denaturation; 34 cycles of 94°C for 20 s, 60°C for 20 s, 65°C for 20 s, 65°C for 5 min. The size of the amplicons was examined by CE using an ABI PRISM® 3730xl genetic analyzer (Applied Biosystems, USA). The actual lengths of VNTR were calibrated to the nearest sizes of corresponding VNTR reported by Keim et al. (5). Each unique allelic combination was defined as an MLVA type (MT).

**Table 2:** Primers used in this study for MLVA-15 analysis of *B. anthracis*.

**VNTR loci**	**Primers**	**Sequences (from 5^**′**^ end to 3^**′**^ end)**
vrrA	vrrA-f-hex	CACAACTACCACCGATGGCACA
	vrrA-r	GCGCGTTTCGTTTGATTCATAC
vrrB1	vrrB1-f-hex	ATAGGTGGTTTTCCGCAAGTTATTC
	vrrB1-r	GATGAGTTTGATAAAGAATAGCCTGTG
vrrB2	vrrB2-f-hex	CACAGGCTATTCTTTATCAAACTCATC
	vrrB2-r	CCCAAGGTGAAGATTGTTGTTGA
vrrC1	vrrC1-f-hex	GAAGCAAGAAAGTGATGTAGTGGAC
	VrrC1-r	CATTTCCTCAAGTGCTACAGGTTC
vrrC2	vrrC2-f-hex	CCAGAAGAAGTGGAACCTGTAGCAC
	VrrC2-r	GTCTTTCCATTAATCGCGCTCTATC
CG3	CG3-f-hex	TGTCGTTTTACTTCTCTCTCCAATAC
	CG3-r	AGTCATTGTTCTGTATAAAGGGCAT
pXO1-aat	pXO1-aat-f-hex	CAATTTATTAACGATCAGATTAAGTTCA
	pXO1-aat- r	TCTAGAATTAGTTGCTTCATAATGGC
pXO2-at	pXO2-at-f-hex	TCATCCTCTTTTAAGTCTTGGGT
	pXO2-at-r	GTGTGATGAACTCCGACGACA
VNTR12	VR12-f-hex	CGTACGAAGTAGAAGTCATTAA
	VR12-r	GCATATAATTGCACCTCATCTAG
VNTR16	VR16-f-hex	CTCTTGAAAATATAAAACGCA
	VR16-r	GAATAATAAGGGTTCTCATGGTAT
VNTR17	VR17-f-hex	TAGGTAAACAAATTTTCGTAATC
	VR17-r	GATCGTACAACAGCAATTATCAT
VNTR19	VR19-f-hex	GTGATGAAATCGGACAAGTTAGGAG
	VR19-r	GAAATATTTTATTAAACATGCTTTCCATCC
VNTR23	VR23-f-hex	TTTAGAAACGTTATCACGCTTA
	VR23-r	GTAATACGTATGGTTCATTCCC
VNTR32	VR32-f-hex	AACTGGATCCAGGAGATTATA
	VR32-r	GAAACAAGAGCAAACCCAAT
VNTR35	VR35-f-hex	AAATAATATGTTCCTTTTGCTG
	VR35-r	GTCCTGAAATAAATGCTGAAT

*B. anthracis, Bacillus anthracis; MLVA, multiple-locus variable-number tandem repeats analysis; VNTR, variable-number tandem repeats*.

### Single-Nucleotide Polymorphism Analysis

The canSNP analysis with 13 markers (A.Br.001, A.Br. 002, A.Br. 003, A.Br. 004, A.Br. 006, A.Br. 007, A.Br. 008, A.Br. 008, B.Br.001, B.Br.002, B.Br.003, B.Br.004, and A/B.Br.001) was performed as described by van Ert et al. (15). Briefly, DNA extracts were processed for CanSNP analysis using 13 TaqMan minor groove binding (MGB) allelic discrimination assays with oligonucleotides and probes as reported by Van Ert et al. (Table 3). The results of CanSNP analysis were compared to the currently reported 12 sublineages or subgroups (13).

**Table 3:** Primers used in this study for SNP analysis of *B. anthracis*.

**SNP group**	**Sequences (from 5^**′**^ end to 3^**′**^ end)**	**Probe sequences (from 5^**′**^ end to 3^**′**^ end)**
A.Br.001	CAAGCGGAACCAAATTTAATCTTT	FAM-ACCGAAACTTGAAGTC - MGB
	TTCACCGTACGTCATTGTATAATACG	VIC-AAACCGAAATTTGAAGTC - MGB
A.Br.002	AACGATACCTAAAATCGATAAAG	FAM-CGCCCAGCCTAA-MGB
	GGCAGAAGGAGCAAGTAATGTT	VIC-CGCCCAACCTAAA -MGB
A.Br.003	GCTACTGTCATTGTATAAAAACCTCCTTT	FAM-TACCTCAAGCTTAATTC- MGB
	CGCTTGCCAAGCTTTTTTTC	VIC-CTACCTCAAACTTAATTC- MGB
A.Br.004	CCGATACCAGTAAACGACGACAT	FAM-TTGGAATGCCCCTAAT- MGB
	CTGGAATTGGTGGAGCTATGGA	VIC-CTTTGGAATGTCCCTAAT- MGB
A.Br.006	CCGGAAATTGCTATTAGAACGAA	FAM-CCATACGCCTAGTGC-MGB
	TCCCAATCTAGCGTTTTTAAGTTCA	VIC-CATCGCCTCGTGCA-MGB
A.Br.007	TTGGTAACGAGACGATAAACTGAATAA	FAM-CATCCTTACATTCAGCT-MGB
	GCCTTGGATTGGCGATTG	VIC-CCATCCTTATATTCAGCTC -MGB
A.Br.008	TTCGCAACTACGCTATACGTTTTAGAT	FAM-ATAATTCTTCGCCGCTTG-MGB
	CAAACGGTGAAAAAGTTACAAATATACG	VIC-ATTCTTCTCCGCTTGTT-MGB
A.Br.009	GGCAATCGGCCACTGTTT	FAM-CGGCTTTGCTTGC-MGB
	GGGTTTCTACTGTGTATGTTGTTAATAAAAAG	VIC-CGGCTTTACTTGCATC-MGB
B.Br.001	TGCATGCTTCTTCTTACAGAGTAGTTAAT	FAM-CGATACCTTCTTATCCTC-MGB
	CGGTCATAAAAGAAATCGGTACAA	VIC-CGATACCTTCTTATCTTC-MGB
B.Br.002	TGTTGCACCTTCTGTGTTCGTT	FAM-CGTTACTGCTGTTCC-MGB
	GTAGTGGCTTCACCGAATGGA	VIC-AACGTTACTTCTGTTCCT-MGB
B.Br.003	CATTTATTCGCATAGAAGCAGATGA	FAM-ACATATCCACTTCACG- MGB
	TGTGCCATCAAATAACTCTTTCTCAA	VIC-CATATCCGCTTCACG-MGB
B.Br.004	GAAGTTAAGTATCAACCAGCAGAAGAAA	FAM-CTTTACTTCTATCATCCC-MGB
	CCGCCGCCTTGAGCTT	VIC-CTTTACTTCTACCATCCC-MGB
A/B.Br.001	GAAGGTCTCCAATTTGGATTTAAAAT	FAM-TTTTATTTAGAAGATAGCGGC-MGB
	CGTGTGAACCTTTCGGTAAATAGTC	VIC-TTTATTTAGGAGATAGCGGC -MGB

*B. anthracis, Bacillus anthracis; SNP, single-nucleotide polymorphism*.

### Cluster Analysis

The MLVA profiles of the two strains isolated in this study, strains isolated in Zhijin County reported in our previous study, and strains previously described in the literature were used for clustering analysis with the BioNumerics software package (version 5.10, Applied Maths, Belgium). The minimum spanning tree (MST) algorithm was applied to establish an MST to show the phylogenetic pattern.

## Results

### Epidemiological Investigation Results

On September 14, 2016, one cow suddenly died in Wan village of Zhijin County in Guizhou Province, and four villagers participated in butchering the dead cow. On September 16, 2016, one of the four villagers displayed symptoms including fever and pimples and blisters on the arm. The patient took cold medicine (medicine for treating colds), but the medicine did not alleviate the illness. On September 20, the patient was received by the emergency department of Zhijin County Hospital and, based on the symptoms, was primarily diagnosed as having suspected cutaneous anthrax. On September 20, the epidemic investigation staff from Zhijin County CDC reported the epidemic to the Guizhou Provincial CDC and investigated the epidemiological history of the patient. During the investigation, they collected the blood and blister fluid samples and five environmental soil samples from the location where the dead cow was butchered. All samples were subsequently sent to Guizhou Provincial CDC for laboratory detection.

### Conventional Identification Results

Two strains suspected to be *B. anthracis* were isolated, including one isolated from the patient's blood sample and the other from the patient's blister sample. The two isolates were identified as *B. anthracis* using a conventional phage lysis test and a penicillin inhibition test (Table 4).

**Table 4:** Background information on the *Bacillus anthracis* isolates from the 2016 epidemic in Zhijin County in Guizhou Province.

**No**.	**Strains**	**Source**	**Prefecture**	**County**	**Year**	**Phage lysis**	**Penicillin inhibition**	**pXO1**	**pXO2**
1	2016001	Human skin	Bijie	Zhijin	2016	+	+	+	+
2	2016002	Human blister	Bijie	Zhijin	2016	+	+	+	+
3	Positive control	/	/	/	/	+	+	+	+

*+, positive*.

### PCR Identification Results

The PCR detection results further showed that the two isolates suspected to be *B. anthracis* from the epidemic, which were primarily identified using the abovementioned conventional methods (Table 4), were both positive for the PA and CAP genes.

### Results of Multiple-Locus Variable-Number Tandem Repeats Analysis

**Figure 1:**
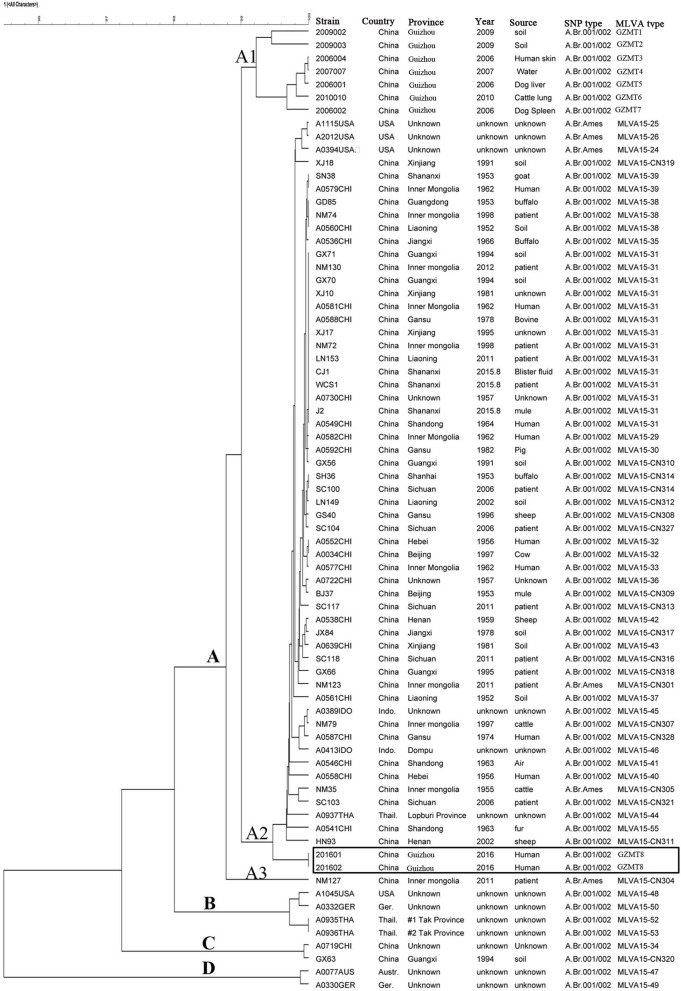
Relationship between the *Bacillus anthracis* isolates based on multiple-locus variable-number tandem repeats (VNTRs) analysis (MLVA). The two *B. anthracis* isolates from a human anthrax epidemic in Guizhou Province were analyzed by MLVA based on 15 VNTR loci. The dendrogram based on the MLVA profiles of isolates from the epidemic from Zhijin and other regions in Guizhou and strains from other countries in the world was constructed using unweighted pair group method with arithmetic mean (UPGMA). The canSNP typing results are also shown in the cluster tree.

MLVA-15 VNTR loci were conducted to analyze the two isolates of *B. anthracis*. The results showed the two isolates have the same MLVA-15 profile (312-228-158-584-524-157-128-139-112-271-383-92-194-564-112). Based on their unique MLVA profile, the two *B. anthracis* isolates belonged to one MT, which was designated as GZGT8. Further, the MLVA profile based on 15 loci was distinct from the 55 profiles of *B. anthracis* collected from other regions in Guizhou Province and representative strains from other countries (Figure 1).

### Single-Nucleotide Polymorphism Analysis Results

CanSNP analysis with 13 markers was conducted to genotype the two isolates of *B. anthracis* isolated in Guizhou Province. The single nucleotide profile for the two *B. anthracis* isolates generated from the 13 SNP loci is T-A-G-C-A-T-T-A-T-G-G-T-A.

### Genetic Relationship Based on Clustering Analysis

The MLVA cluster tree, which was based on the MLVA data of the five isolates and the 55 profiles of isolates from other regions of Guizhou Province and isolates from other countries, showed high diversity (Figure 1). All of the MLVA profiles, including the GZMT8 profile of the two isolates from Zhijin County and the 55 MTs of isolates from other regions of Guizhou Province and representative strains from other countries, can be grossly divided into clusters A, B, C, and D. Most of the representative strains from China were clustered in cluster A, while only two isolates from China were clustered in cluster C. Cluster A can further be divided into the A1, A2, and A3 subclusters. The two isolates from this study belonged to subcluster A3, which can be further divided into several clades, in which the two isolates from Guizhou province grouped into one clade with a coefficient of similarity of 100%. Strains from the United States, Germany, and Thailand were clustered in cluster B, and strains from Australia and Germany were clustered in cluster D.

### Minimum Spanning Tree

**Figure 2:**
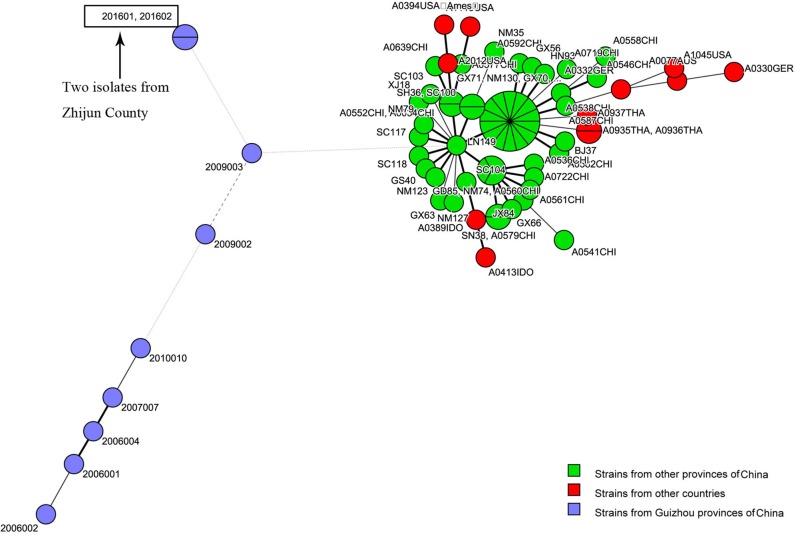
Genetic relationships based on a minimum spanning tree (MST) of the two multiple-locus variable-number tandem repeats (VNTRs) analysis (MLVA) profiles of *Bacillus anthracis* isolates from the anthrax epidemic in Zhijin County and other regions in Guizhou Province. Each circle corresponds to an MLVA profile. The size of the circle is proportional to the number of isolates.

The MST, which is based on the MLVA profiles of the two isolates and 55 MLVA profiles of representative isolates from other regions of Guizhou Province and strains from other countries, showed that the strains from Guizhou Province, including the two strains isolated in this study, were closely distributed (blue circles), while the representative strains from other provinces of China (orange circles) and strains from other countries in the world (red circles) were closely distributed in the MST (Figure 2).

## Discussion

Anthrax is a worldwide zoonosis, and sporadic anthrax cases have been reported occasionally in all countries of the world (16, 17). Some farming regions in China are in naturally infectious areas prone to anthrax outbreaks; livestock are commonly infected by *B. anthracis* during farming, so many recurrent animal anthrax cases are reported (17).

Guizhou Province is a historical center of anthrax in China, and sporadic human anthrax epidemics have happened frequently in Guizhou Province in recent years (9, 10, 18, 19). In this study, a case of suspected anthrax case was reported to the Guizhou Provincial CDC in September 2016. Two suspected *B. anthracis* strains were isolated from the blood and blister samples collected from the patient in this epidemic. Conventional identification, which included Gram staining, a phage lysis test, and a penicillin inhibition test for anthrax diagnosis, identified the two isolates as *B. anthracis* (Table 4).

*B. anthracis* belongs to the *B. cereus* group, which includes *B. cereus, B. thuringiensis, Bacillus mycoides, Bacillus pseudomycoides, Bacillus cytotoxicus*, and *B. anthracis* (20). *B. anthracis* spores can keep stable for decades. The ecology and evolution may be greatly influenced by this longevity. The evolutionary change rate of *B. anthacis* may be reduced by the dormant stage which may result in the extremely homogeneous feature of *B. anthracis* populations (5, 21).

It has been revealed that the chromosome of *B. anthracis* resembles those of non-*B. anthracis* members in group of the *B. cereus*, whereas the two plasmids were identical to plasmids pXO1 and pXO2, which represent the anthrax virulence (22). The anthrax toxins encoded by plasmid pXO1 and capsules encoded by the plasmid pXO2 are the major virulence factors (23). To confirm whether the two isolates were virulent strains, we used PCR to detect the PA and CAP genes, the target genes for the pXO1 and pXO2 plasmids, respectively. The PCR detection results revealed that the two isolates of suspected *B. anthracis* from the epidemic (Table 4) were positive for the PA and CAP genes, which indicates that the two isolates are virulent *B. anthracis* strains.

Previous studies have verified the lack of molecular polymorphisms of *B. anthracis*, which result in the difficulty to subtype it (5, 21, 24, 25). Commonly used genotype methods, including pulsed-field gel electrophoresis (PFGE) and multiple-locus sequence typing (MLST), are not able to distinguish closely related species. By using PFGE and MLST genotyping, many strains within a species show identical patterns (5). Two molecular approaches, MLVA-15 and whole-genome SNP discovery and analysis, have greatly enhanced the identification of genetic markers that help to establish the phylogenetic relationships among *B. anthracis* isolates (5, 15). For the present study, in order to further demonstrate that the infection was caused by *B. anthracis* and to reveal the genetic relationship of the two isolates with strains from other regions, we used MLVA-15 and canSNP to genotype the two isolates. MLVA-15 showed that the two *B. anthracis* isolates, based on the unique MLVA profile that belonged to one MT (GZGT8), were distinct from the 55 profiles of *B. anthracis* collected from other regions in Guizhou Province and representative strains from other countries (Figure 1). SNP analysis based on 13 markers showed that the two isolates belonged to the A.BR.001/002 subgroup. The cluster tree and MST indicated that the two isolates were closely related to strains from other regions of Guizhou Province, which suggest that this epidemic was a local epidemic but not imported from other province or other countries.

The field epidemic investigation conducted by the Guizhou Provincial CDC and the Zhijin County CDC showed that a cow, owned by a villager from Wuan Village, died suddenly, and a total of four people from the village participated in butchering the dead animal. Among the four people, one developed a fever, with red pimples and blisters on the arm. The local county doctor diagnosed it as a suspected case of anthrax, and the patient was hospitalized. Aside from the four people who participated in the butchering, there were 13 people who ate the cooked stomach collected from the cow. No other people displaying anthrax-related symptoms were observed, except for the one who participated in butchering the cow. Based on the clinical symptoms, including red papules, pimples, blisters with tissue swelling and infiltrate, skin ulcers with necrosis and black scabs, in combination with the epidemiological history of the patient, the patient was diagnosed with anthrax.

In this epidemic investigation, soil samples from the environment where the dead cattle were butchered were also collected for *B. anthracis* isolation. However, no strains suspected to be *B. anthracis* were isolated, which may be due to the cleaning of the contaminated soil environment with water after butchering, incorrect collection of samples, or distribution of the bacteria in the soil environment. To avoid spread of the epidemic due to possible environmental contamination, several rounds of disinfection of the environment were performed by the local CDC. The soil samples for *B. anthracis* isolation were collected after disinfection, and no *B. anthracis* was detected from the soil samples. During the investigation, investigators attempted to collect meat samples from the dead cow for *B. anthracis* isolation and identification, but the meat had been sold to other people who subsequently resold the meat in the market. Therefore, no meat samples were successfully collected for *B. anthracis* detection.

Although no *B. anthracis* was successfully isolated from the soil environment and the dead cow, *B. anthracis* was isolated from the blood and skin blister fluid samples. Both conventional and molecular techniques identified the two bacterial strains as virulent *B. anthracis*, which provided the etiological basis for the control and prevention of the epidemic. Additionally, as anthrax is zoonotic and the patient had a history of contact with the sick cow, it can be determined that the patient's case was one of cutaneous anthrax caused by butchering a sick cow. Because the spores are resistant to many disinfectants, disinfection by drying and radiation, the spores can remain surviving for decades in soil (26). The local husbandry and health department of the government should strengthen its surveillance of animal anthrax and environmental contamination caused by *B. anthracis* and take measures to disinfect the environment of the natural foci, which could reduce the risk of broader epidemic of anthrax. From a bioterrorism and biowarfare perspective, *B. anthracis* could be used as the agent of bioterrorism and biowarfare. The government should attach extremely great importance on the storage, transportation, and handling of *B. anthracis*. All of the activities have to meet the requirement of the biosafety regulation, which could reduce the risk of bioterrorism and biowarfare.

## Conclusion

The results from the molecular investigation suggested that the epidemic was caused by *B. anthracis* infection, which provided an etiologic basis for the diagnosis of patients and the investigation of this epidemic. Additionally, the two *B. anthracis* isolates recovered from the epidemic were genetically distinct from isolates from other regions of China and other countries. Our results will not only aid in understanding disease origins and transmission patterns but also facilitate the development of various measures to control anthrax.

## Data Availability Statement

The SNP data generated has been uploaded to the European Variation Archive (EVA), Project: PRJEB36719, Analyses: ERZ1300463. Other raw data supporting the conclusions of this article will be made available by the authors, without undue reservation, to any qualified researcher.

## Ethics Statement

Ethical approval was not required in accordance with institutional guidelines and national legislation. All data have been anonymized, so written informed consent was not required.

## Author Contributions

SL conceived and designed the experiments, wrote and edited the manuscript. QM and YL performed the experiments. SL, QM, and HC analyzed the data. SL, GY, and GT performed the epidemiological investigation. SL, GT, and DW contributed reagents, materials, and analysis tools.

### Conflict of Interest

The authors declare that the research was conducted in the absence of any commercial or financial relationships that could be construed as a potential conflict of interest.

## Abbreviations

CDC: Center for Disease Control and Prevention
canSNP: canonical single-nucleotide polymorphisms
MLVA: multiple-locus variable-number tandem repeat analysis
CAP: capsule
PA: anti-protective antigen
PCR: polymerase chain reaction
SNR: single nucleotide repeat
VNTR: variable-number tandem repeat.
